# A physiologically inspired hybrid CPG/Reflex controller for cycling simulations that generalizes to walking

**DOI:** 10.1371/journal.pcbi.1013494

**Published:** 2025-09-12

**Authors:** Giacomo Severini, David Muñoz

**Affiliations:** 1 School of Electrical and Electronic Engineering, University College Dublin, Dublin, Ireland; 2 Insight Center for Data Analytics, University College Dublin, Dublin, Ireland; McGill University, CANADA

## Abstract

Predictive simulations based on explicit, physiologically inspired, control policies, can be used to test theories on motor control and to evaluate the effect of interventions on the different components of control. Several control architectures have been proposed for simulating locomotor tasks, based on fully feedback, reflex-based, controllers, or on feedforward architectures mimicking the Central Pattern Generators. Hybrid architectures integrating both feedback and feedforward components represent a viable alternative to fully feedback or feedforward controllers. Current literature on controller-based simulations almost exclusively presents task-specific controllers that do not generalize across different tasks. The task-specificity of current controllers limits the generalizability of the neurophysiological principles behind such controllers. Here we propose a hybrid controller for predictive simulations of cycling where the feedforward component is based on a well-known theoretical model, the Unit Burst Generation model, and the feedback component includes a limited set of reflex pathways, expected to be active during steady cycling. We show that this controller can simulate physiological stationary cycling patterns at different desired speeds and seat heights. We also show that the controller can generalize to walking behaviors by just adding an additional control component for accounting balance needs. The controller here proposed, although simple in design, represent an instance of physiologically inspired generalizable controller for cyclical lower limb tasks.

## Author summary

Predictive simulations allow to synthesize human movements and their associated biomechanical quantities without using experimental data. Such simulations can help understand how humans may perform movement tasks in different scenarios and could provide useful information in fields like rehabilitation. One of the methodologies used for developing predictive simulations is based on creating models of the neural control architectures that generate the activation of the muscles. In this work we propose the first neural control architecture for cycling behaviors. We show that our architecture can be used to develop predictive simulations of cycling at different speeds and that the associated biomechanical quantitites are consistent with experimental data. Moreover, we show that our control architecture can also replicate walking behaviors with minimal modifications.

## 1. Introduction

Predictive neuromechanical simulations (PNS) have been proposed as a way to test motor control theories and as a mean to predict the potential effects of therapy, assistive devices and modifications in the biomechanical characteristics of a person [1,2].

PNS are usually obtained from solving an optimal control problem to find the control parameters that allow a biomechanical model to complete a desired task. The biomechanical model represents the human body with an accuracy and physiological constraints that depends on the topic of the research. The solution to the optimal control problem is found through the minimization of the value of a task-specific cost function. Two approaches have been mainly used for both structuring and solving such optimal control problems, direct control and policy-based control. In the former case the optimal control problems are solved to directly find the states and excitations of the model. The most common method to derive the trajectory of the states and excitations is Direct Collocation (DC) [3]. In control-policy based PNS the optimal control problem is set to find the parameters of a control policy (e.g., the gains of a set of reflexes [4], or the timing and amplitude of a set of oscillators [5]). This control policy consists of a set of physiologically-inspired ruling equations which derive the activations of the actuators of the model. In this approach, the parameters of the controller are usually found using evolution strategies [6].

While humans perform a vast repertoir of lower limb tasks through the same physiological structures and similar control strategies [7–9] we still lack comprehensive control policies that can be used to simulate the execution of different lower limb tasks. As an example, cycling and walking are two tasks that share similarities in control and can possibly be represented by similar control policies.

Literature presents several examples of predictive simulation studies of cycling. A few seminal studies have used dynamic optimization and genetic algorithms to estimate timing and magnitude of torques or muscular activation that can replicate cycling behaviors [10–12]. Recent studies employ direct control through DC to investigate different aspects of cycling optimality [13–18], either by tracking experimental data or in fully predictive scenarios. To our knowledge, only few previous works have evaluated the use of control policies for cycling simulations [19,20]. However, these controllers are based on simple rules (e.g., predetermined phasing patterns for the activations of the muscles), leaving the full potential of this approach unexplored.

Control policy-based approaches, on the other hand have been widely used to investigate the underlying control of gait. These policies are based on assumptions on neural control of gait and can be classified into reflex-based, Central Pattern Generator (CPG)-based, or hybrid CPG-reflexes approaches. Each of these approaches can give successful simulations of physiological gait. Reflex-based controllers [4,21,22] implement feedback reflex networks to drive motion strictly according to sensory input. CPG-based architectures [5] implement central pattern generators [23] as feedforward control that efficiently organizes the model activity. Hybrid controllers, which combine both feedback and feedforward control components, have long been proposed as viable solutions for locomotion [24,25]. More recently, hybrid controllers incorporating physiologically inspired reflex-based architectures [22] have been shown to exhibit advantageous properties of both fully reflexive and fully CPG approaches [26,27]. However, as introduced before, a recurrent problem of control policies for gait is the impossibility to generalize the controller to other motor tasks. Recent efforts in developing new architectures for PNS have offered breakthroughs in control generalization. For example, a recent work [28] was able to integrate standing up and walking behaviors in a single simulation by attaching sequentially two dedicated controllers. We recently used a general modular architecture to replicate a transition from standing to walking [27].

**Fig 1:**
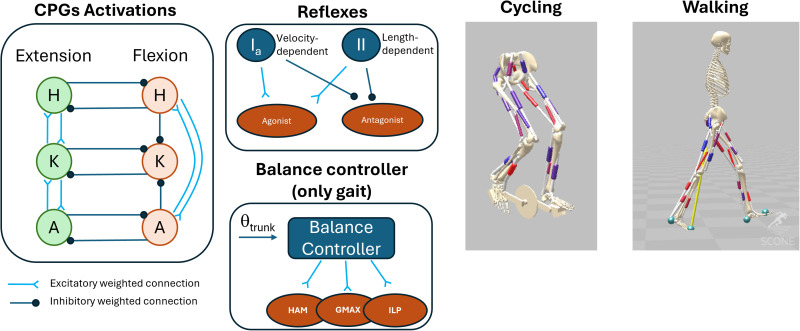
General overview of the controller and the models. The input to the motorneurons is given by the combined contributions of the CPGs, Reflexes and, for the walking simulations only, the balance controller. The CPGs activations are designed based on the UBG model proposed by Grillner [23]. There are six activation unit for the extensor and flexor muscles of the hip **(H)**, knee (K) and ankle **(A)**. The activations are determined by equations 3 and 4 and are modulated by the activations of the other CPGs as shown in figure. The reflex architecture is constituted by Ia and II receptors that excite the same muscles and inhibit their antagonist based on velocity and length feedback. The balance controller is a PD controller that directly modulates the activity of the hip flexors and extensors based on the trunk angle [21]. All inhibitory and excitatory connections have associated weights, determined during the optimization process. The control components have been applied to both a cycling and a walking model, which are structurally identical but for the presence of the gear and pedals (which are linked to the feet) in the cycling model and the contact surfaces in the walking model.

**Fig 2:**
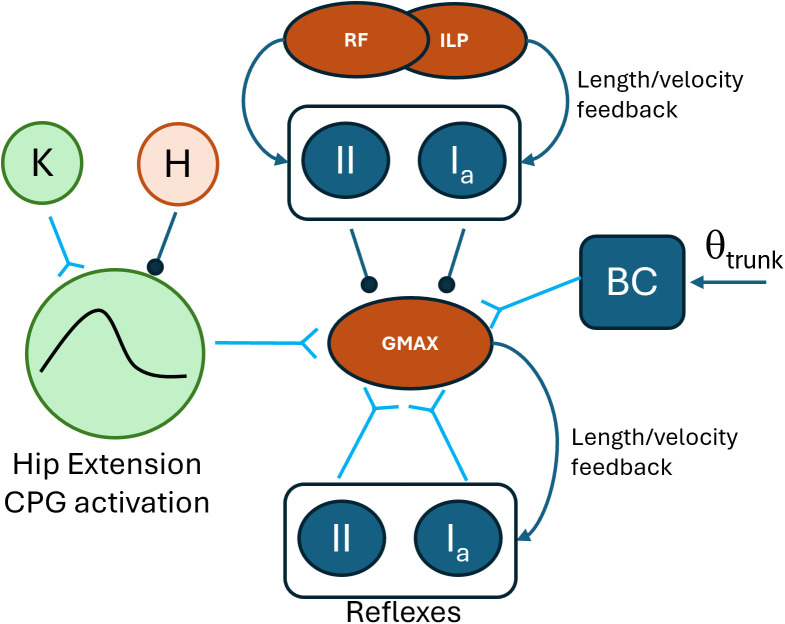
Breakdown of all excitatory and inhibitory connections to a muscle. The figure presents all inhibitory and excitatory connections to a muscle (GMAX taken here as example). The activation of the muscles is driven by the corresponding CPG activation (hip extension in this case), excitatory Ia and II reflex modulation by the agonists and the muscle itself, inhibitory Ia and II reflex modulation from the antagonists (ILP and RF in this case) and, for gait, the contribution of the balance PD controller. The CPG activation is excited and inhibited by other CPGs as per the UBG scheme (K extension and H flexion in this case, see Fig 1). All inhibitory and excitatory connections have associated weights, determined during the optimization process.

Here we propose a hybrid neural controller architecture which combines a reflex network and CPGs (**Figs 1 and 2**) and that can be used to simulate both cycling and walking behaviors. The feedback loops represented by the reflexes are physiologically inspired while the CPGs provide a feedforward control which is plausible and it is based on previous successful results [26,29]. The feedforward component of the controller is inspired by the Unit Burst Generator model proposed by Grillner [30]. In this scheme, CPGs are independent units of bursting activity intertwined in a network. The output of the units depends on their intrinsic properties but also on the configuration of the network. The overall controller aims at representing the first instance of a physiologically-inspired neural controller for stationary cycling, that can also generalize to similar tasks such as gait. Exploring the underlying neural control of cycling and its similarity to gait is crucial as cycling is an exercise often used in the rehabilitation setting. Developing a physiologically plausible controller may help in the development of simulations that can estimate the effects of specific cycling-based intervention while discriminating between effects of the intervention on the feedforward and feedback components of control. The simulations obtained optimizing the parameters of the controller we propose have resulted in the replication of cycling behaviours at different pedaling speeds and seat height, presenting metrics that are generally consistent with human data. Additionally, we show the potential of this architecture to generalize, with minimal task-specific modifications, to walking behaviors. After showing that this controller can achieve realistic cycling and walking behaviors, we conclude that the combination of reflex and UBG networks offer new and interesting possibilities to develop general control architectures for repetitive lower limbs movements. These architectures are intended both to advance our understanding of motor control and to enable control-level personalization in simulations, thereby improving the representation of individual impairments.

## Results

**Fig 3:**
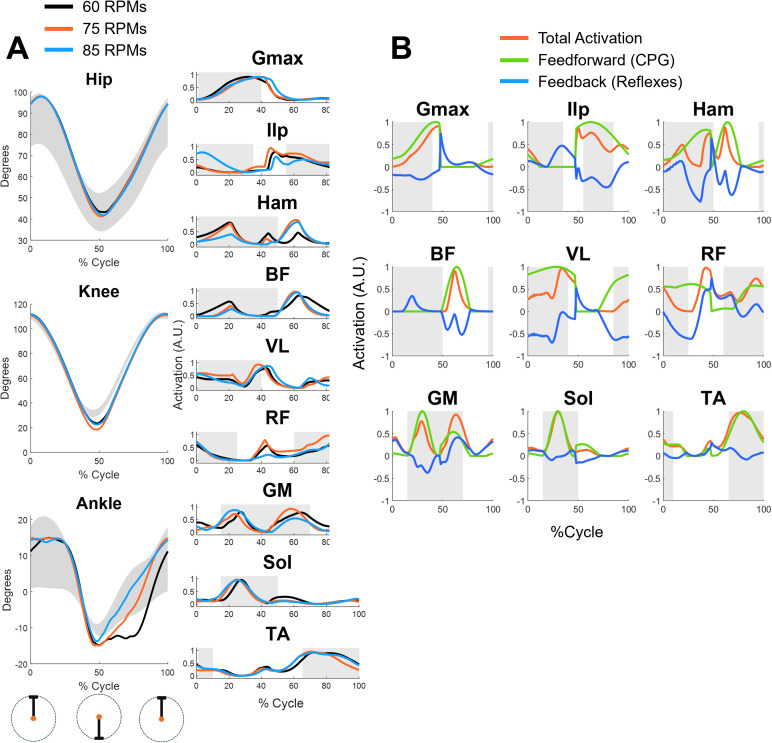
Results for the cycling simulations at different speeds. Panel A shows the joint angles (left) and muscular activations (right) obtained from the cycling simulations at 60 (black), 75 (orange) and 85 (blue) RPMs. The 0% and 100% of the cycle represent the top pedal position during the cycle. The shaded area in the joint angles represent normative joint angles during cycling while the shaded areas in the muscular activations represent the normative timing of muscular activations (derived from [13] and [31]). Panel B shows the relative contribution of CPGs (green) and reflexes (blue) to the overall activation of the muscle (orange) for the 75 rpms speed.

We used an optimization procedure based on the Covariance Matrix Adaptation Evolution Strategy (CMA-ES) to find the parameters of the controller that can achieve successful cycling and walking simulations in different conditions. The biomechanical model was almost identical between the two tasks (see Methods for details) and consisted of 9 actuators per leg: gluteus maximus (GMAX), iliopsoas (ILP), hamstrings (HAM), biceps femoris (BF), vastus lateralis (VL), rectus femoris (RF), gastroecnemius medialis (GM), soleus (SOL) and tibialis anterior (TA). The proposed hybrid controller was able to achieve both cycling and walking behaviors, with biomechanics and muscular activations consistent with those recorded experimentally in both tasks. The joint angles obtained in the simulations mostly agree with the experimental results (**Fig 3A**). For cycling, the optimization aimed at finding parameters that optimized a cost function minimizing effort, while matching a desired speed (60, 75 or 85 revolutions per minute, RPMs) and penalizing non physiological joint range of motions, loads and muscular inactivation (see Methods).

We observed a slight over-flexion of the hip at the beginning of the cycle and a slight over-extension of the knee at the dead bottom part of the cycle (50% of the cycle). The ankle angle was consistent with the average values observed experimentally, although an excessive plantarflexion was observed in the second half of the cycle (upstroke) for the 60 and 75 RPMs simulations. These differences at the knee and ankle may be explained, other than from possible limitations of the controller, by the fact that the simulations are in 2D. In fact, in the experimental data the oscillation on the transverse plane may contribute to the joint angle patterns in the sagittal plane.

Similarly to previous works [21,26], the muscular activations were compared with on-off dynamics (timing of muscular activations) observed experimentally during unperturbed walking [32] and stationary cycling [13,31] (a direct comparison with experimental EMG data from [13] is provided in S2 Fig). We observed a general accordance between the simulated and experimental timing of activation of the muscles. HAM and BF displayed patterns which are not entirely physiological. Both presented additional activations during the second half of the cycle. Although consistent with a pulling motion, these activations are not normally observed during standard, non-efficient cycling [33]. We also observed peaks in the activity of the RF, VL and Ilp close to the dead bottom center of the cycle, which are not usually observed during standard cycling. Only minimal differences in muscular activations across the different speeds were observed, with the simulations at 75 and 85 RPMs characterized by a more prominent activity of the HAM during the second half of the cycle. To get a better insight on the roles of the different components of the controller, we analyzed the different contributions of feedforward, CPG-mediated and feedback, reflex-mediated components to the overall activations of the muscles (**Fig 3B**). Reflex activity was mostly characterized by inhibitory reflexes limiting the CPG-mediated activation of the muscles, especially during the downstroke phase of cycling (0–50% of cycle). This was observed mostly for the muscles acting on the knee and hip, like HAM, VL and RF muscles, while the contribution of the reflexes was negligible in the muscles controlling the ankle. Positive reflex contribution was observed mostly at the beginning of leg retraction phase (around 50% of the cycle). For most muscles, the overall timing of activation was dictated by the CPG activity, with the reflexes modulating, mostly in inhibition, the final muscular activation level. This observation suggests that the optimization procedure leads to a CPG behavior consistent with a pulling motion (BF and HAM) during the leg retraction phase of cycling.

**Fig 4:**
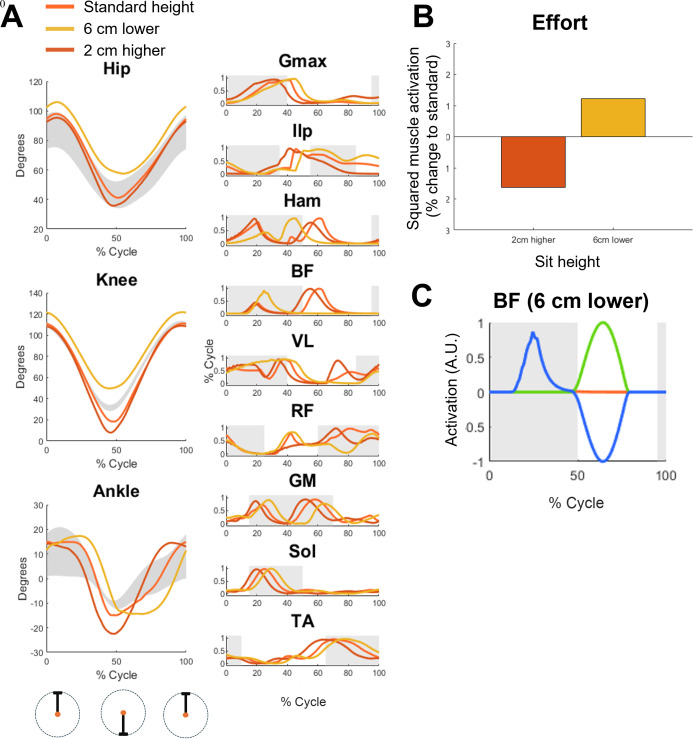
Results for the cycling simulations at different seat heights. Panel A shows the joint angles (left) and muscular activations (right) obtained from the cycling simulations at standard (orange), 6 cm lower (yellow) and 2 cm higher (red) seat height. All simulations were carried out at 75 rpms using the parameters found in the standard seat height optimization. The 0% and 100% of the cycle represent the top pedal position during the cycle. The shaded area in the joint angles represent normative joint angles during cycling while the shaded areas in the muscular activations represent the normative timing of muscular activations (derived from [13] and [31]). Panel B shows the % change, with respect to the simulations at standard seat height, of the effort, calculated as the average squared muscular activation across all muscles. Panel C shows the relative contribution of CPGs (green) and reflexes (blue) to the overall activation of the BF muscle (orange) for the 6 cm lower condition.

We then analyzed whether a controller optimized on the optimal seat height [13]could replicate cycling behaviors at different seat heights. One of the aims of this analysis was also to characterize whether the biomechanical characteristics we observe during the cycling motion depend on control or on the posture of the model. We observed (**Fig 4**) that the controller optimized for optimal seat height can replicate also higher (2 cm) and lower seat heights (2, 4 and 6 cm, data shown only for 6 cm for simplicity). As expected, seat height influences the joint angles by introducing a bias and slightly increasing (for higher seat height) or decreasing (for lower seat height) the range of motions of the hip, knee and ankle. The musclar activations are similar across consitions, with appreciable differences observed mostly for the lowest seat height. Here we observe a pattern of activation of the BF and HAM that is consistent with standard, non-optimal cycling. Interestingly, in the BF the muscular activation during the pulling phase is completely absent. When analyzing the different contributions of reflexes and CPGs to this behavior we notice that the CPG-related pulling is still present but is completely inhibited by antagonist reflexes.

For gait, the optimization aimed at finding the parameters minimizing a cost function aiming at achieving a stable gait at a self-selected speed above 1m/s, while minimizing effort, head acceleration and penalizing non-physiological range of motions and muscular inactivations.

**Fig 5:**
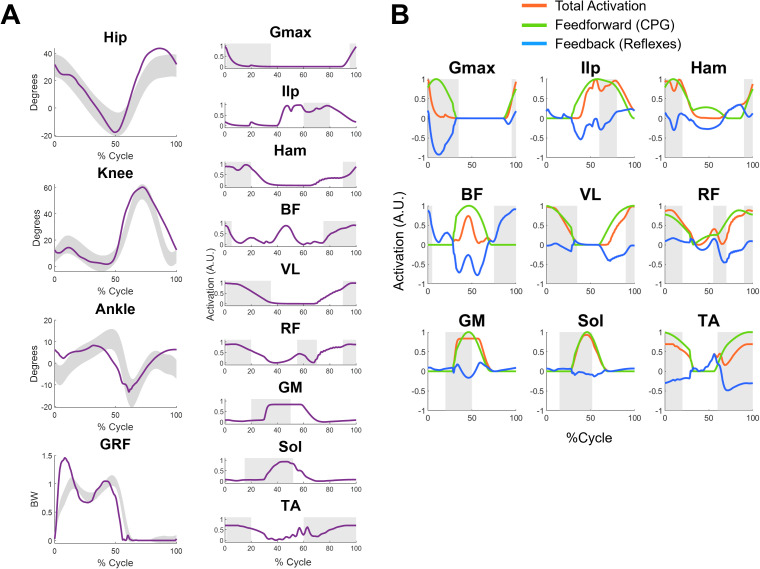
Results for the walking simulations at different speeds. Panel A shows the joint angles and vertical ground reaction forces (left) and muscular activations (right) obtained from the walking simulations. The optimization procedure achieved a “preferred” gait speed of 1.4 m/s. The shaded area in the joint angles represent normative joint angles during walking, while the shaded areas in the muscular activations represent the normative timing of muscular activations (derived from [32]). Panel B shows the relative contribution of CPGs (green) and reflexes (blue) to the overall activation of the muscle (orange). For the Ilp, Gmax and Ham muscles the reflex contribution include also the contributions of the balance controller.

When optimized for gait the controller achieved a gait speed of 1.46 m/s. The simulations showed physiologically consistent biomechanics and muscular activations, with some small differences (**Fig 5A**). We observed an increment in the hip flexion during late swing, coupled with a slight increased knee flexion and ankle plantarflexion. The ankle pattern lacks a transition from dorsiflexion to platarflexion during late swing, indicative of a lack of foot preparation for landing. This likely translated in the increased ankle dorsiflexion during heel-strike. We also observed an anticipated ankle plantarflexion during late stance.

The vertical ground reaction forces were consistent with physiological data but characterized by a high peak during load acceptance. This also could be indicative of a lack of foot preparation during landing. The patterns of muscular activations are generally consistent with the timing observed in experimental studies [32], with some differences. Such is the case of Ilp, which activation is prolonged, the activation of BF, which presents an unexpected peak during late stance, or delayed activations of the ankle extensors. When analyzing the contribution of feedback and feedforward control for the gait optimizations (**Fig 5B**), we found that, similarly to cycling, the activity of the muscles acting on the ankle were mostly driven by the CPG activity. For all the other muscles, the biggest contribution of the reflexes is once again observed in inhibition and downregulation of the activity of the CPGs, with the notable exception of the BF muscles, where substantial excitatory reflex activity is observed during late swing and early stance. The reflex activity of the ILP, Gmax and HAM also includes the activations due to the balance controller, which cannot be directly separated from the other reflex activity acting on those muscles. However, also for these muscles, the timing of activation is mostly dictated by the CPG activity, with reflexes modulating the amplitude in the ILP and HAM and reducing the activation timing for the Gmax.

**Fig 6:**
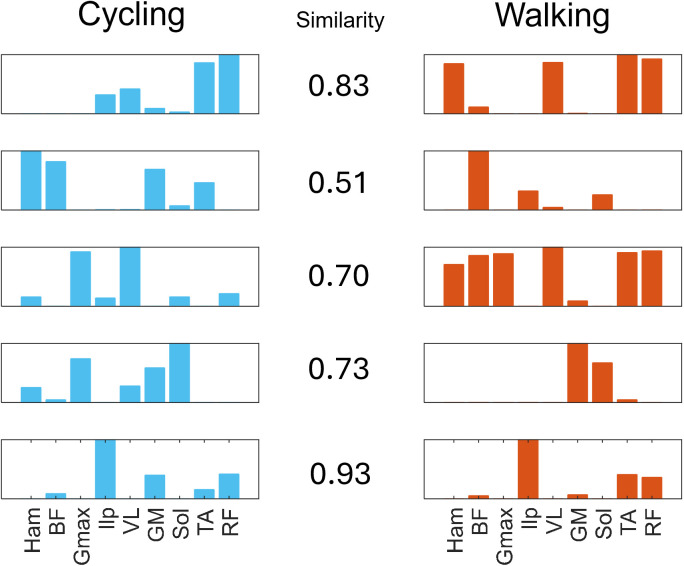
Synergies comparison. Muscle synergies extracted for cycling (left, blue) and walking (right, red), matched by similarity (calculated as the cosine product between the weight modules).

Finally, to investigate the similarity in control between gait and cycling, we extracted the muscle synergies from the simulations of both tasks (**Fig 6**). We used an objective criterion based on the Akaike information criterion for the selection of the number of synergies [34] which found five synergies for both cycling and walking. Some of the synergies showed a high similarity between the two scenarios. Both tasks presented an almost identical synergy. For example, S5 presented a similarity, as indicated by the cosine product, of 0.93 characterized by the activity of the Ilp, TA and RF. The same three muscles mostly characterized another synergy, S1, that was remarkably similar between the two tasks (similarity of 0.83). In opposition, this synergy is presenting an additional activation of the ILP for cycling and the Ham for walking. S3 presented a similarity of 0.7 between tasks, arising from the co-activation of the Gmax and VL in both tasks. However, the same synergy also couples the activation of the knee flexors and the TA and RF in walking. S4 represents, for both tasks with a similarity of 0.73, the activity of the ankle plantarflexors, although in cycling we also observe a co-activation of the Gmax. Finally, S2 is the most dissimilar module between the two tasks (similarity of 0.51), sharing only the activity of the BF. Overall, the biggest differences across the modules appear to be related mostly to the activity of the hip flexors and extensors. These muscles are the only which do not share the same control architecture between the two tasks, given the presence of the balance controller for walking.

## 2. Discussion

In this study we proposed a physiologically plausible hybrid CPG-reflexes neural controller architecture. We showed that, after task-specific optimization of the control parameters, the controller can achieve realistic biomechanics and muscular activations during both cycling and walking simulations. The control architecture we propose is one of few in the field of predictive simulations of movement that can generalize across different tasks [27,28].

Our work is the first to propose a physiologically plausible neural controller for control-policy based cycling simulations. Recently, Delp and colleagues developed a trajectory optimization-based, data-informed simulation of cycling using MOCO [13,35]. In their work, the authors used experimental kinematic and kinetic data to estimate joint, muscle forces and muscular activation in a sample of healthy individuals. Although both ours and their works showcase the potential of PNS for cycling, they also highlight, in our opinion, some conceptual limitations of PNS in general. In both works, the simulations result in muscular activation for the HAM and BF during upstroke. Although optimal for the task, this dynamic is normally not observed physiologically during standard cycling, but is instead more common in mechanically optimal cycling [33] The HAM and BF, in fact, are normally activated in the last phase of downstroke during cycling, while only trained cyclists, by employing a pulling strategy, display hamstrings activation in the first phases of the upstroke. The hamstrings, as knee flexors, when activated during down-stroke, de-facto resist the motion, while their activation during upstroke would provide positive torque towards the cycling motion. This “paradoxical” [36] activation of the hamstrings during cycling is well known and most likely deriving from their role in controlling final foot position during walking. Predictive simulations of cycling, on the other hand, appear to discover the task-optimal activations. Here we show that the optimization of the CPG parameters lead to CPG activations for the HAM and BF that are consistent with mechanically optimal cycling. However, as recently pointed out in a review on predictive modeling [2], human neuromuscular control, although well explained by the solution to optimal control problems, is inherently suboptimal. Our results confirm that simulating cycling using optimal control further highlights the underlying suboptimality of physiological control. Locomotion and cycling share similar control characteristics. Locomotion, however, is a key human behavior that has been adapted through evolution [37] and whose basic control patterns are observable from early age [38]. Cycling, on the other hand, is a relatively recent behavior. Given its similarities with gait, cycling most likely employs similar control strategies [39]. These strategies, although mostly optimal for gait, may be suboptimal in cycling (e.g., the activity of the hamstrings during downstroke). Even though optimal control techniques have shown remarkable potential when simulating human behaviour, task-specific suboptimality needs to be accounted for. Suboptimality can be represented by either adding constrains at the control level (e.g., forcing specific synergetic activations) or by designing ad-hoc cost functions (e.g., penalizing hamstring activations during upstroke).

In our results we show that, in both cycling and walking simulations, the CPGs are the main contributors to the timing and magnitude of muscular activations, with the reflexes mostly modulating the activity (**Figs 3–5**). The role of reflexes in a hybrid controller of gait has been comprehensively described by Di Russo and colleagues [26,40]. In that work, the authors developed a detailed reflex architecture which uses interneurons regulating the activation of disynaptic reflexes. They also include, differently than this work, reflex pathways controlling excessive force production and muscular activations. Here, given the high number of parameters to be optimized in the CPG part of the controller, we decided to employ a simpler reflex architecture. Such architecture only consisted of monosynaptic equivalents of Ia and II reflex pathways, which regulate activation of the muscles depending on length and velocity feedback. Moreover, we speculated that since we planned to simulate behaviors at submaximal force production condition, where reflexes controlling excessive force production and muscular activations would ideally not be activated, those components may not be needed.

The results on the different contribution of CPGs and reflexes, paired with additional simulation we performed on cycling without the reflexive components (see S1 Fig) show that the cycling behavior can be achieved using only the CPGs. Similar results were obtained in the past also for gait using CPG-based control architectures [5,41]. This opens the question on whether a simple reflex architecture like the one we proposed is necessary during cycling simulation.

The literature on reflexes in gait models, either as sole control components or as sub-components of a hybrid controller, suggest the necessity of including fast feedback pathways in gait simulations [21,22,26,42,43]. Similarly, the reflex pathways that we both included and excluded in our controller are physiologically known to be active during walking [44–47]. Based on the similarity in control between cycling and gait and on the previous argument on how, evolutively, cycling may have control derived from walking, it is fair to assume then that reflex pathways are active also during cycling. This has been confirmed also in experimental studies [48,49]. Thus, the potential limitation of the reflex architecture we here propose could be its simplicity rather than its presence.

Our results show that our controller, which was originally designed for cycling, generalizes also to simulating walking behaviors, after adding a control component accounting for balance [21]. The main limitation of the controller during gait appears to depend on the lack of foot preparation for landing during the swing phase. This is a problem that is common to other hybrid controllers [26,27] that do not include control components specific for this function during late swing. In our simulations the ankle remains dorsiflexed right before heel strike, rather than returning to a neutral position. We speculate that including a dynamic balance element in the cost function may lead to better foot-adjustment during swing in our architecture. Dynamic balance has been shown to be a major driver of short-term adjustments in gait [50,51]. Proper foot adjustment, in fact, minimizes impact forces and sudden fluctuations in the velocity and acceleration of the center of mass and in the margin of stability.

Cycling and walking have been shown to share similarities in their associated muscle synergies [8,33]. Here we factorized the muscular activity of both tasks into 5 synergies using an objective algorithm [34]. We show that 4 out of 5 of the synergy modules extracted during the two tasks share remarkable similarities. The synergies themselves, compare mostly favourably with experimental synergies observed in literature. For example, S1C, including RF and TA, and S2C, which includes the knee flexors and the Gmax, have been observed in several other experimental works on cycling [8,31,33]. S1W is associated with the activity of the Vasti and has also been observed in experimental studies [7,8,52] while S2W does not present evident similarity with experimentally observed modules. S3C is characterized by the Gmax and VL. However, while experimental studies have shown synergies in cycling where these two muscles contribute together, there is no observation of synergies mostly constituted by these two muscles. The S3W synergy shows activity of the TA, RF and knee flexors. Being active during late swing/early stance, this synergy contributes to response to impact and landing preparation, that, has we already argued, is deficient in our results. In experimental works, these functions are usually disentangled into two synergies [9]. S4C and S4W are the dorsiflexion synergy which is often observed experimentally in both cycling and walking [7,33,53]. Finally, S5C and S5W, which are the most similar between the two tasks, include the hip flexor and the RF. This synergy is commonly observed in walking and has been observed also in cycling, when the hip flexors are included in the recordings [31].

Most of the differences in the synergy modules between the two simulated tasks is accounted by differences in the activation of the Ham (S1-3C and S1-3W), BF (S3C and S3W) and Gmax (S4C and S4W). This could be expected as these are the only muscles with differences in the control architecture, given the presence of the balance controller for gait. The balance controller is a PD controller active during stance that regulates the activity of the hip muscles based on feedback on the trunk position. The presence of feedback components regulating the angle of the trunk with respect to the pelvis during walking is plausible, although their exact architecture is not known. Thus, we cannot exclude that a physiological balance controller would be constituted by feedback-based circuits mapping directly at the level of the CPGs rather than on the single muscles. Synergies have often been indicated as the building blocks of movement and believed to reflect networks of spinal interneurons coordinating the activity of muscles in group [54]. Our results show that physiologically plausible synergies arise from the coordinated activity of flexion and extension CPGs and reflexes. In the upper limbs, the link between synergies and neural control may be more straightforward to analyze, as synergies likely reflect coordination patterns encoded at the spinal level that are mostly independent of reflexes and cyclic activation. In contrast, our results suggest that synergies in the lower limbs, derived from EMG decomposition, may obscure the contributions of multiple neural control pathways involved in generating muscle activity.

Predictive simulations have long been proposed as a mean to test the effect of therapies or changes in characteristics of impairment [1]. The few works that have tested the used of PNS of impairment were mostly based on DC-based simulative architectures [55–57], although recently, control-policy based methods have been used on biomechanical models simulating impairments [58]. While DC-based simulations show remarkable potential in the simulation of steady-state behaviors, the incorporation of feedback is still being investigated [59]. Currently proposed DC-based simulations offer little possibility of personalization in the control strategy, thus limiting the possibility of making patient-specific models. Control personalization ìn DC-based simulations has, so far, only been modeled as patient-specific muscle synergies, or through the modification of the neuromechanical characteristics of the biomechanical and muscle model to account for deficits such as spasticity or weakness [55,60]. Here we show that synergies can arise from the activity of CPGs and reflexes, and do not appear to differentiate between the different control components. Conditions such as stroke can affect both feedback and feedforward components of control, differently. Stroke survivors often exhibit hyperreflexia, that can be modeled in the feedback component of a control-policy based architecture (as shown in a recent work on Cerebral Palsy [61]) but also in the simplified control of the feedforward component. Given this rationale, control-policy based predictive simulations offer, at least theoretically, greater possibility of control personalization and could be effectively used to model the effect that different intervention may have on the different control components, separately. The drawback, however, of policy-based simulation is the impossibility to validate the control rules underlying the simulation as accurate [62]. For this reason, developing control policy that are fully based on experimentally validated principles, although not a validation, represent the safer approach. Finally, we here demonstrate that a common control architecture can be optimized to achieve two different tasks. Future works will have to focus on expanding on the generalizability of the proposed architecture (e.g., increasing the number of muscles, or including an analog to the balance controller in cycling) to possibly test whether it is possible to obtain different tasks through a common optimization procedure.

### Limitations

Our work presents some limitations. Both the biomechanical model and the controller are simple when compared with physiology. The reflex architecture is simple with respect to some recent models [21,26]. The balance controller used in this study is relatively simple and based on a previous work. In future studies, a more specialized balance controller, potentially integrated with the central pattern generator (CPG) architecture, could be considered. Our CPG implementation does not account for phase resetting. We decided not to include phase resetting to avoid introducing additional complexity to the optimization process, as the present study focused exclusively on unperturbed walking and cycling. Moreover, while implementation of phase resetting in gait is clear for gait, it is less clear for cycling. However, we acknowledge that incorporating phase resetting is necessary to test the controller in more natural and dynamic scenarios beyond unperturbed cycling and locomotion. Moreover, we completely omitted possible inhibitory and excitatory connections, both in the reflexes and the CPGs, between the two legs. Additionally, our simulations are constrained to a 2D scenario. While this simplicity limits the comparison of our results with experimental ones, it is dictated by the necessity of testing the feasibility of the proposed approach. Although the controller presents a reductive reflex architecture, and it was applied to a simple biomechanical model already tested [21,26], it needed a multi-stage optimization of more than 100 parameters. Thus, optimization time, difficulties in convergence and risk of genetic drift [63] increased. Another limitation of our work, shared with similar works in literature, is the empirical nature of the cost functions that we employed. The weights associated with the cost function elements were selected empirically based on preliminary results. A more substantial analysis of the elements of the different cost functions and their associated weight, also considering different weighting techniques [64], would likely improve our result.

## 3. Methods

### 3.1 The biomechanical model

The cycling and gait simulations were based on the same 2-D biomechanical model, applied to the two different scenarios (**Fig 1**). The model was derived from the one developed by Delp and colleagues [65] which was also used in similar works [21,26]. Seven segments represent the human body in this biomechanical model. Feet, shanks, thighs and pelvis are present in the cycling scenario where pelvis tilt and translations were fixed, and a gear-crank-pedals assembly was attached to the model. The cranks can rotate around the gear and the pedals can rotate around the crank in response to forces exerted by the foot in the sagittal and coronal planes. For simplicity, the joint connecting the feet to the pedals was locked so that no movement was possible between these two segments. This implementation of the model presents 9 DoFs, consisting of gear and pedals rotation, and hip, knee and ankle frontal rotation. The height of the seat in the cycling model was set to match the criteria for seat height used in Clancy and colleagues [13]. In the gait version of the model, feet, shanks, thighs and HAT (head-arms-torso) are present. The gait model presents 9 DoFs, consisting of pelvis tilt and anteroposterior-longitudinal translations, and hip, knee and ankle frontal rotation. The gait model presented two contact spheres for each foot (positioned at the talus and toes, Fig 1). The model, in both scenarios, present 18 Hill-type muscles (9 per legs): hamstrings (Ham), biceps femoris short head (BF), gluteus maximus (Gmax), iliopsoas (Ilp), vastus medialis (VM), rectus femoris (RF), gastroecnemius medialis (GM), soleus (Sol) and tibialis anterior (TA). The model was implemented in the SCONE software [66] for the Hyfydy simulation engine [65] Geijtenbeek, T. The Hyfydy Simulation Software. 2021, 11. https://hyfydy.com. Hyfydy utilizes a semi-implicit Euler integrator. The time-step of integration *dt* was here set to 0.001 seconds.

### 3.2 The controller

The controller was designed as a hybrid controller composed by components of feedforward and feedback control (**Fig 1**). The feedforward component is based on the Unit Burst Generator model (UBG-model) proposed by Grillner [23]. This model consists of unit CPGs inter-connected in a network. The feedback component of the controller consists of a set of reflexes regulating the muscular activity based on sensory inputs. Particularly, muscle length and velocity feedback reflexes are implemented in the controller. For the walking simulation, a third component, a balance controller, is added. This controller is an additional feedback component which is active during early and late stance. The role of this controller is to control the trunk angle by acting only on the hip muscles. This control has been used in previous models [21,26,42]. For each muscle, the excitation at each instant is the sum of the excitation arising from all CPGs and reflex pathways in which the muscle is involved (**Fig 2**). Hip muscles receive also an excitatory component from the balance controller during the walking simulations.


$$u_{tot-cycling}=\;\sum u_{CPGs}+\sum u_{reflexes}\;$$ (1)

$$u_{tot-walking}=\;\sum u_{CPGs}+\sum u_{reflexes}+u_{balance}\;$$ (2)

where *u*_*tot-cycling*_ and *u*_*tot-walking*_ are the total output of the controller to generate muscular activity for cycling and walking behaviors, respectively, *u*_*CPGs*_ is the output of the UBG-model, and *u*_*reflexes*_ is the output of the reflex network. The component *u*_*balance*_ is the output of the balance controllers, only employed for walking simulations.

### 3.3 The central pattern generators

The CPG architecture is based on the previously proposed UBG-model [30]. This model is constituted by unit CPGs which control a set of motoneurons [67]. The units are distributed in a network which consists of inhibitory and excitatory synapses. This coupling between units, besides their intrinsic properties, determines their output.

In the 2D model we employed, each unit is controlled by a rhythmic activation that acts as a clock function. We identified a total of 12 units, 6 per leg, consisting of flexor and extensor units acting on the hip, knee and ankle. In the overall network the different units act as reciprocal inhibitors and/or excitators for the other units, following a scheme precedently proposed in a simulation study [68], and based on experimental observations [69]. In this scheme (**Fig 1**), pairs of flexor and extensor units acting on the same joint provide reciprocal inhibition. Extensors units provide reciprocal excitation to the units of the closest joint (e.g., hip extensors excite knee extensors, which excite both hip and ankle extensors). Flexors units follow a more complex inhibition/excitation pattern, with the hip and ankle units inhibiting the knee unit and exciting each other. The activation of each single unit, as already proposed previously [26], is based on raised cosine function:


$$a_{unit}(\phi ,\mu ,\sigma )=\;\left\{ \;\;\;\begin{array}{c} \frac{\text{1}}{\text{2}}\left(\text{1}+\text{cos}\left(\frac{\phi -\mu }{\sigma }\pi \right)\right)\;,\;\mu -\sigma \leq \phi \leq \mu +\sigma \\ \text{0}\;otherwise \end{array}\right.\;$$ (3)

where ϕ  is the leg-dependent gait phase, μ is the position of the peak of the activation in the cycle, and σ is the witdth of the activation pattern. The output, a_unit_, is expressed in the form of a discrete bell-shaped waveforms, in opposition to a classical oscillator configuration [70]. Differently by the Aoi’s model of dynamically coupled CPGs [29], where the phase generator for both sides is governed by a differential equation coupling the two legs, here we fixed the coupling between the two legs across the whole simulation. Moreover, while previous implementations [26,29] used the heel-strike and foot-off to reset the phase, we did not add an event-based phase reset. Instead, the phase continuously increases. These choices were made under the assumption that our simulations will replicate steady-state unperturbed cycling and walking, where the coupling of the two legs is always maintained to a delay of half-cycle. Thus, at the beginning of each simulation we set $\phi_{left\;}=\text{0}\;and\;{\;\phi }_{right\;}=\pi .$ The phases are then updated at each instant following the equation:


dϕ= ω·dt (4)

where ω is the angular frequency common to all the 12 oscillators and *dt* is the time-step of the simulation. This equation establishes the phase generator, or clock function, of the system. The activation of each single unit was then calculated, at each time point, based on the UBG architecture as follows:


$$a_{hip/knee/ankle\;extension}=a_{unit}+\sum k_{adj}a_{adj}-{k_{ant}a}_{ant}$$ (5)

$$a_{hip/ankle\;flexion}=a_{unit}+{k_{ankle/hip\;}a}_{ankle/hip}-{k_{ant}a}_{ant}\;$$ (6)

$$a_{knee\;flexion}=a_{unit}-\sum k_{dj}a_{adj}-{k_{ant}a}_{ant}\;$$ (7)

Where the *a* parameters are the outputs of the different unit CPGs and represent the *u*_*CPG*_ values from equation (1) at the current time point. The *k* parameters are weights in the interval [0;1] whose values are set through the optimization process. The suffixes *adj* and *ant* in the *a* and *k* parameters in equations (5–7) mean adjacent and antagonist respectively. For the extension CPGs, each unit has a total output that is the sum of the activation of the unit itself, plus a weighted excitatory contribution coming from the adjacent units and a weighted inhibitory contribution coming from the corresponding antagonist flexion unit. For flexion, hip and ankle receive a weighted excitatory contribution from each other and inhibition from the corresponding extension unit. The flexion knee receives inhibition from all adjacent units and the corresponding extension unit. The contribution of the units to each muscle (*u*_*muscle/CPG*_) is calculated as:


$$u_{muscle/CPG}=\;w_{muscle/CPG}a_{unit}\;$$ (8)

where the *w* parameters are weights regulating the contribution of each muscle associated to a unit. The muscles only receive activity from the units to which they are associated. For example, Gmax only receives the output generated by the hip extension unit, while RF receives output from both the hip flexion and knee extension units. The feedforward component of the controller has a total of 37 parameters to be optimized. Those parameters include the weights *w* and *k*, the values of μ and σ of each CPG, and the general angular frequency of the clock function ω. To minimize the number of parameters to be optimized, we assumed complete symmetry between the two legs and the same values were associated to parameters mapping on the right and left leg.

### 3.4 The reflex architecture

In our control architecture we employed a series of reflex pathways, derived from previous works [26,71] but simplified based on task-related considerations. The reflex architecture we employed include:

a) Ia afferents, providing self-excitation and agonist inhibition to the muscles based on a velocity-dependent response to stretch [47].
b) II afferents providing excitation to the same muscle and inhibition to the antagonist muscle(s) based on muscle length [46].

We did not include Ib afferents and Reshaw cells in our architecture [44,45]. Ib afferents provide same and antagonistic inhibition from the same muscle and its antagonists and are triggered by large forces. Renshaw cells provide same and antagonistic inhibition and are triggered by excessive muscular activations. Here we perform simulations on two tasks, cycling and walking, both performed at a sub-maximal exertion level and optimized on an energy minimization objective. We then assume that, under these task conditions, Ib afferents and Renshaw cells, which are both mechanisms controlling excessive force production, are not expected to contribute significantly. The reflexes were all modeled as monosynaptic reflexes, differently from the comprehensive interneuron-mediated network presented by Di Russo [26]. This choice was made to minimize the number of parameters to be optimized. The excitations relative to the reflexes were calculated following equations 9 and 10 for Ia and II afferents respectively:


$$u_{v}=k_{rv}v(t-t_{D})$$ (9)

$$u_{l}=k_{rl}l(t-t_{D})$$ (10)

where *k*_*rv*_ and *k*_*rl*_ are the gains of the reflexes, *v* and *l* are muscle velocity and length, and *t*_*D*_ is the muscle-specific time delay, 5, 10 or 20 ms, determined from previous studies on reflex-based controllers [21]. The gains *k* are set during the optimization procedure and are positive for excitatory reflexes and negative for inhibitory ones. Each muscle is characterized by 2 excitatory gains relative to Ia and II, and one or two inhibitory gains to its antagonists. In total, 46 gains (18 excitatory, 28 inhibitory) were optimized during both gait and cycling. Weights were also assumed symmetrical between the two legs to minimize the number of parameters to be optimized.

### 3.5 The balance controller

In the walking simulations, due to the need for stability, a balance controller was added to the architecture. This component consists of the trunk control policy originally implemented by Ong and colleagues [21]. The balance controller is implemented based on a state-machine and is only active during early and late stance. The objective of this new component is the regulation of the trunk angle by acting on the Ilp, Gmax and Ham muscles. The controller is a proportional derivative controller and its output follows the equation:


$$u_{balance}=\;k_{p}\left(\theta (t-t_{D})-\theta_{\text{0}}\right)+k_{v}\dot{\theta }$$ (11)

where *k*_*p*_ and *k*_*v*_ are the proportional and derivative gains, θ is the trunk angle (to which a time delay equal to 5 ms is applied) and $\theta_{\text{0}}$ is the desired value for the trunk angle. A total of 9 parameters (a set of *k*_*p*_, *k*_*v*_ and $\theta_{\text{0}}$ for Ilp, Gmax and Ham each) were optimized for this controller.

### 3.6 Optimization procedures

The cycling and walking simulations had 101 and 110 parameters to be optimized, respectively. The parameters are divided in 18 position and velocity offsets to the initial pose, 37 CPG parameters, 46 reflex parameters, and the additional 9 parameters of the balance controller only present for the gait simulations. The optimization was performed using the Covariance Matrix Adaptation Evolutionary Strategy, implemented on the SCONE software [66]. Due to the number of parameters to be optimized we employed a multi-stage optimization procedure where the parameters found in one stage were used as the initial guess for the following stage.

### 3.7 Cycling task optimization

The optimization for cycling was divided in two stages. A first stage found the initial cycling solutions that matched the target speed of pedaling while limiting the range of motion of the ankle. The second stage, employing the solution of the first stage as initial guess, aimed at finding parameters that minimized muscle effort and the loads at joints, while limiting their range of motion to physiological values. Additionally, solutions involving inactive muscles were penalized during optimization, as such patterns, while potentially optimal in a computational sense, are not physiologically plausible for the muscles and tasks considered. The cost function for the stage 1 was then:


$$J_{cycling-\;stage\;\text{1}}=J_{speed}+J_{ankleROM}$$ (12)

where the element *J*_*speed*_ minimizes the difference between the desired speed (set at 60, 75 or 85 RPMs) and the actual speed achieved by the model during the simulation, while the element *J*_*ankleROM*_ restricts the ankle range of the solution to a physiologically observed range of [-15 deg; 15 deg]. The cost function for the stage 2 was:


$$J_{cycling-\;stage\;\text{2}}=J_{speed}+{J_{effort}+J}_{jointROMs}+J_{jointLoads}+J_{activation}$$ (13)

where element *J*_*speed*_ is equivalent to the one used in stage 1; the element *J*_*effort*_ minimizes the sum of the squared muscle activations of all the muscles; element *J*_*jointROMs*_ is the extension of element *J*_*ankleROM*_ of stage 1 to all the joints, restricting the ranges of the hip, knee and ankle joints to [25 deg; 95 deg], [25 deg;120 deg] and [-15 deg; 15 deg] respectively; element *J*_*jointloads*_ minimizes the loads at all the joints, as shown to be beneficial in previous cycling simulations [13]; element J_activation_ penalizes muscular activations below 0.1 in a range [0; 1]. This latter element aims at avoiding solutions where one or more muscles are inactive. A low muscle activation profile is likely to happen during the optimization process as the control architecture employed in this study includes several inhibitory pathways between multiple sub-blocks of control. The weights of each element of both cost functions (eq. 12 and 13) were set empirically. The different elements of the cost function were not normalized. For this reason weights are not presented as they would not provide an intuitive indication of the importance of each element in the cost function. Each optimization stage was run for a maximum of 10,000 generations, but optimization was stopped when improvement in fitness were below 1e-5.

### 3.8 Walking task optimization

The optimization for walking was divided in three stages, in a scheme similar to the one proposed by Di Russo and colleagues [26]. The stage 1 was not used to achieve a gait behavior. Instead, the objective is initializing the parameters to achieve segment movements and muscular activations similar to those of a physiological gait behavior. The biomechanics and muscular activations to imitate were obtained by running the optimized reflex-based controller developed by Ong and colleagues [21]. The cost function for stage 1 was then:


$$J_{walking-\;stage\;\text{1}}=J_{imitation}$$ (14)

where *J*_*imitiation*_ minimizes the difference of the muscular activations obtained from our model respect to the simulation results derived from the Ong controller. The stage 2 of the optimization aimed at obtaining a realistic gait behavior, using the parameters obtained in stage 1. The cost function for stage 2 was:


$$J_{walking-\;stage\;\text{2}}=J_{gait}+J_{effort}+J_{limit}+J_{head}$$ (15)

where *J*_*gait*_ penalizes solutions outside the set velocity range (1.5 ± 0.1 m/s) and solutions where the model falls. *J*_*effort*_ penalizes the metabolic cost of walking following the methodology developed by Uchida and colleagues [72]. *J*_*limit*_ minimizes the joint limit torques at the knee and ankle, and *J*_*head*_ minimizes the head acceleration in the vertical and horizontal directions. Finally, the optimization in stage 3 is used to discard solutions with inactive muscles and the cost function is:


$$J_{walking-\;stage\;\text{3}}=J_{gait}+J_{effort}+J_{limit}+J_{head}+J_{activation}+\;J_{GRF}$$ (16)

where the first four elements of the cost function are equivalent to stage 2. The *J*_*activation*_ element penalizes muscular activations below 0.1 in a range [0; 1], as in the cycling optimization. Finally, the *J*_*GRF*_ element penalizes peak values of vertical ground reaction force (GRF) exceeding 1.5 the body weight of the model. Also in this case the weights of each element of the cost functions (eq. 14–16) were set empirically and the different elements of the cost function were not normalized.

### 3.9 Simulations

For the cycling scenario, we performed three sets of simulations. For each set of simulations, we ran ten independent optimizations for stage one, then the result with the best convergence was used as initial guess for stage two. The quality of the convergence was based on the fitness calculated from the cost function. Stage two consisted of five optimization processes. The results presented are the best solution among the five optimizations (although difference in final fitness across the best and worst optimization was always < 10%). The three sets consisted of simulation scenarios performed using the full controller (CPG and reflexes) at three different target speeds of 60, 75 and 85 RPMs. S1 Table shows the parameters found for the best optimization at 60 and 75 RPMs. The speed was modulated by changing the element $J_{speed}$ in equations 12 and 13. The aim of these simulations was to test the consistency of the results across different speeds. We then used the parameters found in the 75 RPMs optimization to run additional simulations changing the hight of the seat for the model (results shown for +2 and -6 cm with respect to optimal seat height). To better understand the roles of CPGs during cycling, we performed an additional set of cycling simulations at 75 RPMs with a controller consisting only of the CPG component, without reflexes (results shown in S1 Table).

For the walking scenario, we ran a single set of simulations, to evaluate the capacity of the controller to generalize to a different motor task. We ran ten independent optimization processes for stage one and stage two, and five optimiation processes for stage three.

### 3.10 Validation

To evaluate the ability of our controller to replicate physiological cycling and gait, we compared biomechanical metrics in both tasks with those recorded in existing accessible datasets. The metrics consisted of the angular positions of the joints, and the muscular patterns of activation. Specifically, for cycling, we used the dataset used in Clancy and colleagues [13] to have the comparison of the kinematics. The dataset was recorded from 16 healthy individuals cycling on a stationary bike at self-selected speeds ranging from 77 to 92 RPMs. The muscular activations were compared to the activation timing observed in experimental cycling setups similar to our simulation [31,33]. For walking, we used the dataset recorded by Van Criekinge and colleagues [32] for both, kinematics and muscle activations. The dataset was recorded from 138 healthy individuals walking at self-selected speed.

### 3.11 Muscle synergies analysis

Previous works in the literature have reported on the similarity between the muscle synergies extracted during walking and cycling [8,33]. We here compared the synergies extracted from our cycling and walking simulations. Our objective is to assess whether the muscular activations obtained by applying the same control architecture to two different tasks result in similar synergy modules. We extracted synergies from the muscular activations derived from the simulations using the standard non-negative matrix factorization algorithm [73]. A previously validated objective methodology was used to derive the optimal number of synergies for each task [34]. The similarity between the synergy modules was calculated using the cosine product [74].

## Supporting information

S1 Fig Results for the cycling simulations with and without reflexes. Joint angles (left) and muscular activations (right) obtained from the cycling simulations at 75RPMs with (orange) and without (green) the reflexes in the controller. The 0% and 100% of the cycle represent the top pedal position during the cycle. The shaded area in the joint angles represent normative joint angles during cycling while the shaded areas in the muscular activations represent the normative timing of muscular activations (derived from [13] and [31]).
(DOCX)

S2 Fig Direct comparison between simulated activations and experimental EMG. Each plot shows in grey the average ± standard deviation of experimental EMGs recorded by Clancy et al. 2023, and in orange the muscular activation of one simulation at 75 RPMs.
(DOCX)

S1 Table Optimized reflexes and CPG parameters for cycling at 60 and 75 RPMs. (DOCX)

S1 Video Video of the cycling simulation. The video shows an example of cycling motion deriving from the last stage of the optimization process.
(MP4)

S2 Video Video of the walking simulation. The video shows an example of walking motion deriving from the last stage of the optimization process.
(MP4)
