# Supplementary material for: A physiologically inspired hybrid CPG/Reflex controller for cycling simulations that generalizes to walking
Source: PLoS Comput Biol. 2025 Sep 12;21(9):e1013494. doi: 10.1371/journal.pcbi.1013494 (PMC12445551; doi:10.1371/journal.pcbi.1013494)
Supplement: S1 Fig — Joint angles (left) and muscular activations (right) obtained from the cycling simulations at 75RPMs with (orange) and without (green) the reflexes in the controller. The 0% and 100% of the cycle represent the top pedal position during the cycle. The shaded area in the joint angles represent normative joint angles during cycling while the shaded areas in the muscular activations represent the normative timing of muscular activations (derived from [13] and [31]). (DOCX) [file pcbi.1013494.s001.docx]

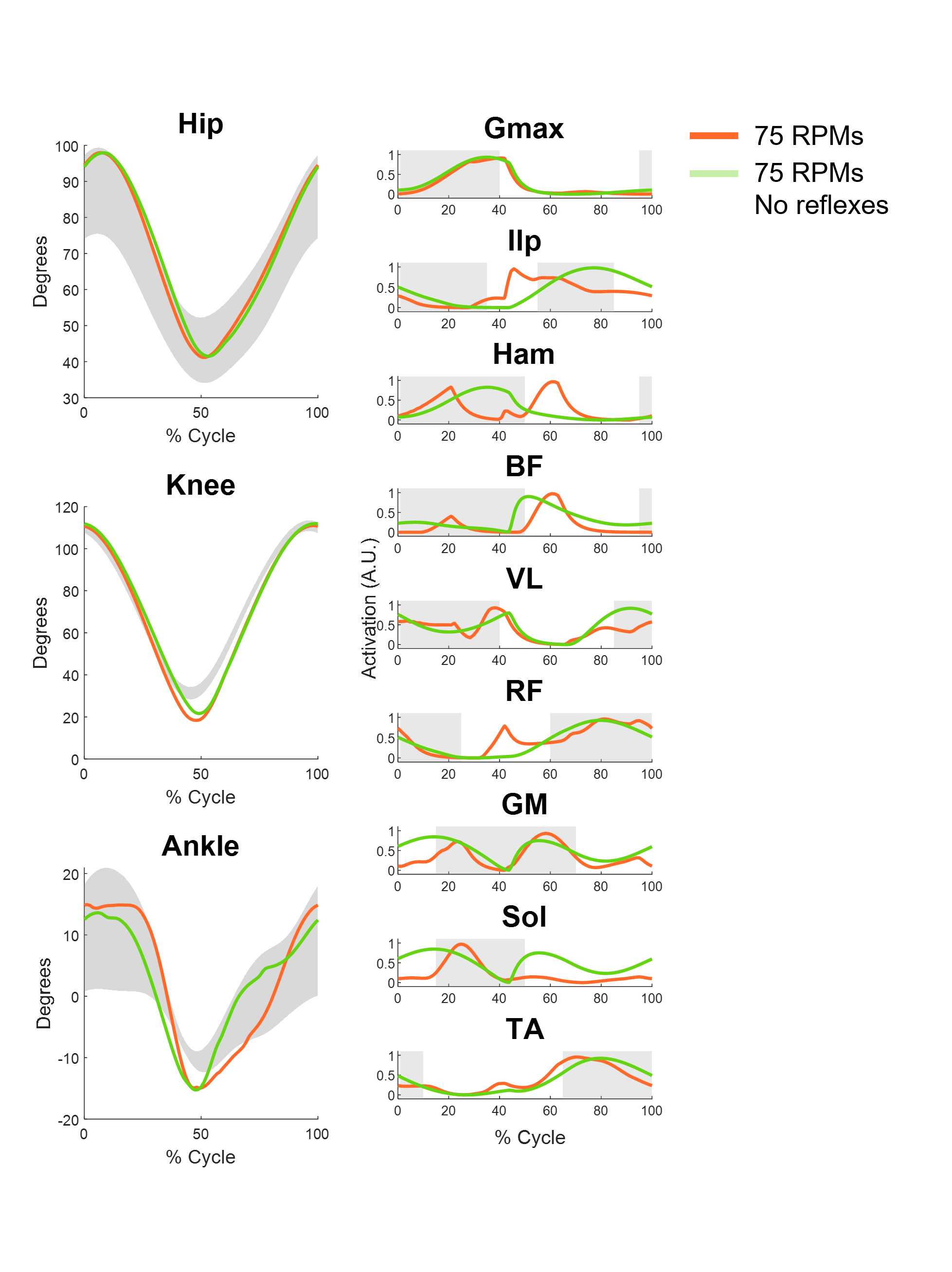


**Figure S1. Results for the cycling simulations with and without reflexes.** Joint angles (left) and muscular activations (right) obtained from the cycling simulations at 75RPMs with (orange) and without (green) the reflexes in the controller. The 0% and 100% of the cycle represent the top pedal position during the cycle. The shaded area in the joint angles represent normative joint angles during cycling while the shaded areas in the muscular activations represent the normative timing of muscular activations (derived from [10] and [28]).
