# Supplementary material for: A physiologically inspired hybrid CPG/Reflex controller for cycling simulations that generalizes to walking
Source: PLoS Comput Biol. 2025 Sep 12;21(9):e1013494. doi: 10.1371/journal.pcbi.1013494 (PMC12445551; doi:10.1371/journal.pcbi.1013494)
Supplement: S2 Fig — Each plot shows in grey the average ± standard deviation of experimental EMGs recorded by Clancy et al. 2023, and in orange the muscular activation of one simulation at 75 RPMs. (DOCX) [file pcbi.1013494.s002.docx]

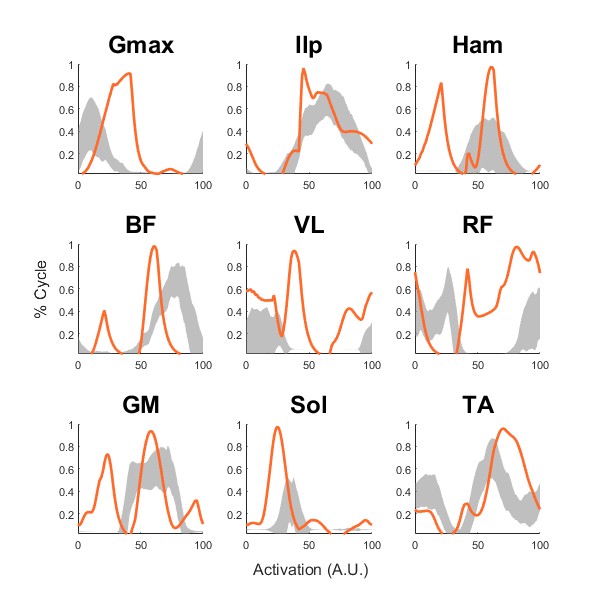


**Figure S2. Direct comparison between simulated activations and experimental EMG.** Each plot shows in grey the average ± standard deviation of experimental EMGs recorded by Clancy et al. 2023, and in orange the muscular activation of one simulation at 75 RPMs.
