# Supplementary material for: A physiologically inspired hybrid CPG/Reflex controller for cycling simulations that generalizes to walking
Source: PLoS Comput Biol. 2025 Sep 12;21(9):e1013494. doi: 10.1371/journal.pcbi.1013494 (PMC12445551; doi:10.1371/journal.pcbi.1013494)
Supplement: S1 Table — (DOCX) [file pcbi.1013494.s003.docx]

**Table S1.** Optimized reflexes and CPG parameters for cycling at 60 and 75 RPMs.

| **Reflex Parameters** |  |  |
| --- | --- | --- |
| Parameter | value 75 RPMs | value 60 RPMs |
| Reflexes.glut_max.KL | 0.10002294 | 0.12752641 |
| Reflexes.glut_max.KV | 0.36549079 | 0.3222279 |
| Reflexes.iliopsoas.KL | 0.28280776 | 0.16849459 |
| Reflexes.iliopsoas.KV | 0.062999056 | 0.000428415 |
| Reflexes.vasti.KL | 0.099615296 | 0.13267657 |
| Reflexes.vasti.KV | 0.097927095 | 0.11530088 |
| Reflexes.rect_fem.KL | 0.44177918 | 0.45392381 |
| Reflexes.rect_fem.KV | 0.056973178 | 0.000375053 |
| Reflexes.hamstrings.KL | 0.58582951 | 0.58669945 |
| Reflexes.hamstrings.KV | 0.30360695 | 0.37422882 |
| Reflexes.bifemsh.KL | 0.74471815 | 0.87473218 |
| Reflexes.bifemsh.KV | 0.30634512 | 0.30204576 |
| Reflexes.tib_ant.KL | 0.45980412 | 0.43261713 |
| Reflexes.tib_ant.KV | 0.000497483 | 0.004982186 |
| Reflexes.soleus.KL | 0.37134598 | 0.35623567 |
| Reflexes.soleus.KV | 0.031681426 | 0.033494132 |
| Reflexes.gastroc.KL | 0.38458831 | 0.44818559 |
| Reflexes.gastroc.KV | 0.018860164 | 0.000155888 |
| Reflexes.iliopsoas-glut_max.KL | -0.16227909 | -0.034738378 |
| Reflexes.iliopsoas-glut_max.KV | -0.34571823 | -0.36153609 |
| Reflexes.glut_max-iliopsoas.KL | -0.34849603 | -0.33690956 |
| Reflexes.glut_max-iliopsoas.KV | -0.067084411 | -0.031465211 |
| Reflexes.vasti-hamstrings.KL | -0.003969403 | -0.002509365 |
| Reflexes.vasti-hamstrings.KV | -0.31920338 | -0.40088471 |
| Reflexes.rect_fem-hamstrings.KL | -0.28230784 | -0.37204065 |
| Reflexes.rect_fem-hamstrings.KV | -0.46643721 | -0.20466904 |
| Reflexes.hamstrings-rect_fem.KL | -0.35298259 | -0.34700437 |
| Reflexes.hamstrings-rect_fem.KV | -0.49055526 | -0.43700738 |
| Reflexes.bifemsh-rect_fem.KL | -0.49948391 | -0.37175525 |
| Reflexes.bifemsh-rect_fem.KV | -0.4211512 | -0.43214744 |
| Reflexes.rect_fem-bifemsh.KL | -0.36114333 | -0.49823192 |
| Reflexes.rect_fem-bifemsh.KV | -0.49812696 | -0.34526196 |
| Reflexes.vasti-bifemsh.KL | -0.31436269 | -0.22653195 |
| Reflexes.vasti-bifemsh.KV | -0.058942262 | -0.27631662 |
| Reflexes.hamstrings-vasti.KL | -0.4425208 | -0.37182482 |
| Reflexes.hamstrings-vasti.KV | -0.43383516 | -0.37544497 |
| Reflexes.bifemsh-vasti.KL | -0.3960647 | -0.1921169 |
| Reflexes.bifemsh-vasti.KV | -0.21510699 | -0.080011269 |
| Reflexes.tib_ant-gastroc.KL | -0.06224177 | -0.081544603 |
| Reflexes.tib_ant-gastroc.KV | -0.1540155 | -0.17630542 |
| Reflexes.tib_ant-soleus.KL | -0.28117691 | -0.24571972 |
| Reflexes.tib_ant-soleus.KV | -0.009304972 | -0.040844996 |
| Reflexes.soleus-tib_ant.KL | -0.4468149 | -0.33722125 |
| Reflexes.soleus-tib_ant.KV | -0.16813215 | -0.28682438 |
| Reflexes.gastroc-tib_ant.KL | -0.42745477 | -0.49937729 |
| Reflexes.gastroc-tib_ant.KV | -0.35635672 | -0.42206325 |
|  |  |  |
| **CPG Parameters** |  |  |
| Parameter | value 75 RPMs | value 60 RPMs |
| GainHipFlexionControlateral | 0.20449063 | 0.22716727 |
| GainHipFlexionIpsilateral | 0.27785159 | 0.29966172 |
| GainHipExtensionControlateral | 0.93496925 | 0.591994 |
| GainHipExtensionIpsilateral | 0.29466155 | 0.24495563 |
| GainHipFlexIlp | 0.40109816 | 0.40035777 |
| GainHipFlexRF | 0.40188775 | 0.41219618 |
| GainExtFlexHam | 0.40771932 | 0.48143667 |
| GainExtFlexGmax | 0.85530989 | 0.75294063 |
| GainKneeFlexionControlateral | 0.41834181 | 0.26013822 |
| GainKneeFlexionIpsilateral | 0.12146648 | 0.13896886 |
| GainKneeExtensionControlateral | 0.47539265 | 0.48080954 |
| GainKneeExtensionIpsilateral | 0.29964533 | 0.29974564 |
| GainKneeFlexHam | 0.96516104 | 1.003059 |
| GainKneeFlexBF | 1.1954888 | 1.1980599 |
| GainKneeFlexGM | 0.40031695 | 0.40034116 |
| GainKneeExtVM | 0.40750027 | 0.40846654 |
| GainKneeExtRF | 0.80423306 | 0.70642179 |
| GainAnkleFlexionControlateral | 0.49263783 | 0.40053277 |
| GainAnkleFlexionIpsilateral | 0.26410125 | 0.2752148 |
| GainAnkleExtensionControlateral | 0.29761534 | 0.43312386 |
| GainAnkleExtensionIpsilateral | 0.10009856 | 0.18917773 |
| GainAnkleFlexGM | 0.71937389 | 0.83920139 |
| GainAnkleFlexSol | 1.0930462 | 1.1992642 |
| GainAnkleExtTA | 0.92825155 | 0.95154187 |
| synergyExtL1-local_delay | 0.97638049 | 0.88667384 |
| sigmaExtL1 | 2.3847398 | 2.0177089 |
| synergyFlexL1-local_delay | 0.15123636 | 0.12125436 |
| sigmaFlexL1 | 3.6542252 | 4.6096379 |
| synergyExtL2-local_delay | 0.67835297 | 0.7322249 |
| sigmaExtL2 | 4.6015483 | 4.5984727 |
| synergyFlexL2-local_delay | 0.17660075 | 0.27142119 |
| sigmaFlexL2 | 1.4957167 | 2.2049706 |
| synergyExtL3-local_delay | 0.31691064 | 0.35448536 |
| sigmaExtL3 | 1.6365243 | 1.7316032 |
| synergyFlexL3-local_delay | 0.8264642 | 0.82752821 |
| sigmaFlexL3 | 0.97562845 | 0.80765493 |
| gain_frequencyL | 1.2366206 | 1.0647619 |
